# Visualization of sodium dynamics in the kidney by magnetic resonance imaging in a multi-site study

**DOI:** 10.1016/j.kint.2020.04.056

**Published:** 2020-11

**Authors:** James T. Grist, Frank Riemer, Esben S.S. Hansen, Rasmus S. Tougaard, Mary A. McLean, Joshua Kaggie, Nikolaj Bøgh, Martin J. Graves, Ferdia A. Gallagher, Christoffer Laustsen

**Affiliations:** 1 Department of Radiology, University of Cambridge, Cambridge, UK; 2 MR Research Centre, Department of Clinical Medicine, Aarhus University, Aarhus, Denmark; 3 Department of Cardiology, Aarhus University Hospital, Aarhus, Denmark; 4 Cancer Research UK, Cambridge Institute, University of Cambridge, Cambridge, UK; 5 Department of Radiology, Cambridge University Hospitals NHS Foundation Trust, Cambridge, UK

**Keywords:** MRI, porcine, renal, sodium, volunteer

## Abstract

Sodium magnetic resonance imaging (MRI) is a powerful, non-invasive technique to assess sodium distribution within the kidney. Here we undertook pre-clinical and clinical studies to quantify the corticomedullary sodium gradient in healthy individuals and in a porcine model of diuresis. The results demonstrated that sodium MRI could detect spatial differences in sodium biodistribution across the kidney. The sodium gradient of the kidney changed significantly after diuresis in the pig model and was independent of blood electrolyte measurements. Thus, rapid sodium MRI can be used to dynamically quantify sodium biodistribution in the porcine and human kidney.

## Graphical abstract

**Figure:**
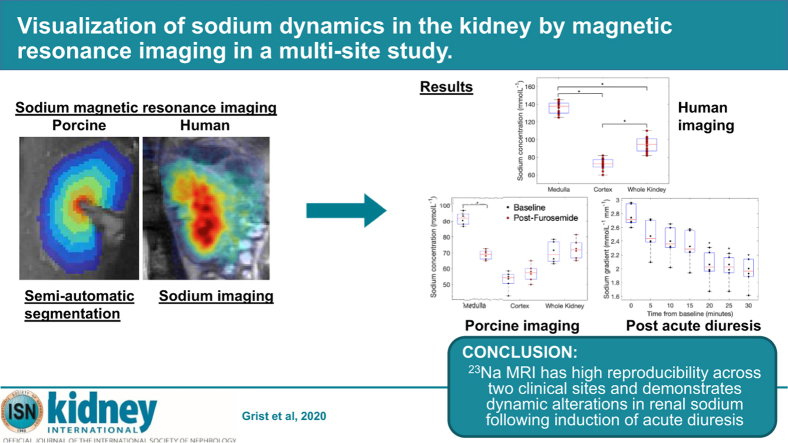

**Translational Statement**

This study demonstrates the use of sodium magnetic resonance imaging (^23^Na-MRI) to quantify the corticomedullary sodium gradient in both the porcine and human kidney and diuretic-induced changes in this gradient in pigs. It shows the potential of using ^23^Na-MRI in conditions that may result in impairment of the sodium gradient, for example, chronic kidney disease, acute renal injury, and pyelonephritis. Renal ^23^Na-MRI may provide new information on the pathogenesis of these conditions as well as provide a functional measure of response to therapy.

Osmolality gradients within the renal parenchyma are essential for normal kidney function. These gradients are partly generated by changes in sodium concentration produced by a number of sodium transporters including the sodium/potassium adenosine triphosphatase transporter,^1^ which increases the concentration of sodium in the interstitium. This high sodium concentration then drives the reabsorption of water into collecting ducts in the renal medulla. Efficient autoregulation of electrolyte reabsorption depends on renal blood flow to maintain oxygen for efficient adenosine triphosphate production within mitochondria in the renal cortex. A decrease in renal blood flow is a common response to cortical injury with a compensatory maintenance of flow to the medulla providing a protective effect against hypoxic injury. Ischemic injury also leads to the inhibition of the sodium/potassium adenosine triphosphatase transporter, further exacerbating cellular injury through accumulation of electrolytes.^2^

Studies have demonstrated that alterations in the intrarenal sodium gradient between the cortex and medulla could be a potential imaging biomarker of renal physiology.^3^^,^^4^ However, these studies require micropuncture or slice section analysis and are not feasible for routine clinical use, are often destructive, and may alter the sodium concentration they are attempting to measure.^5^^,^^6^ Sodium magnetic resonance imaging (^23^Na-MRI) is a noninvasive method to quantify tissue sodium.^7^, ^8^, ^9^ ^23^Na-MRI has been applied to imaging sodium in the kidney and the corticomedullary sodium gradient^10^, ^11^, ^12^, ^13^ and allows for the differentiation of physiologic and pathologic alterations in renal function.^7^^,^^8^ The hypotheses of this study were, first, that the distribution of sodium in the renal system can be reproducibly quantified across 2 clinical sites using ^23^Na-MRI and, second, that the action of furosemide to flatten the corticomedullary sodium gradient can be dynamically measured using the same technique.

## Results

### Renal sodium can be reproducibly imaged and quantified with a 3-dimensional ultrashort echo time acquisition on clinical systems

**Figure 1:**
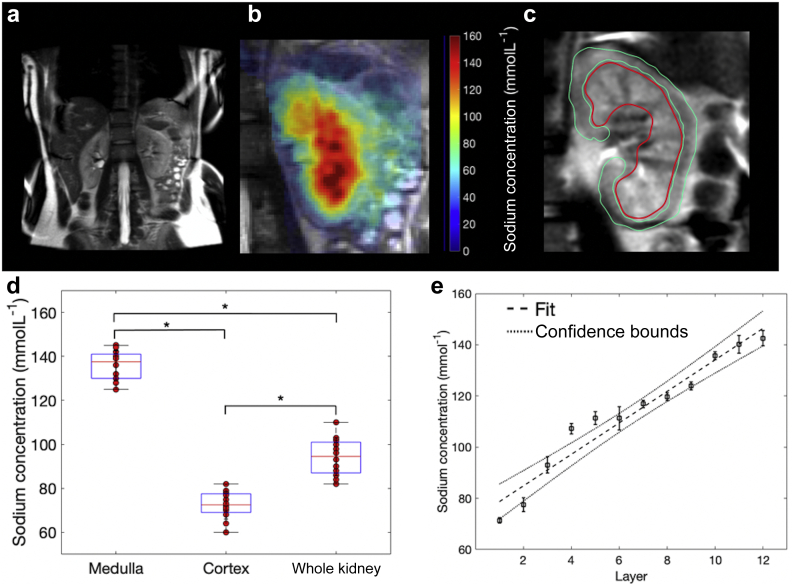
**Sodium imaging of the human kidney**. (**a**) Coronal proton T_2_ magnetic resonance imaging from site A. (**b**) Fused sodium concentration maps and proton imaging from site A. (**c**) Regions of interest showing the cortex (green) and medulla (red). (**d**) Manual segmentation results from the sodium imaging revealing a significant difference between regions of interest. ∗Significant, *P* < 0.05. (**e**) Average corticomedullary sodium gradient from all volunteers across both sites, *R*^2^ = 0.94, *P* < 0.05.

Sodium imaging was successfully acquired in all volunteers, with examples of fused ^1^H, shown in Figure 1a, and ^23^Na concentration imaging, shown in Figure 1b. Segmentation of the kidney into regions of interest (ROIs) is shown in Figure 1c. There were no significant differences in the total sodium concentrations calculated from the segmented medulla, cortex, or whole kidney sodium between sites A and B (site A, 137 ± 2 mmolL^–1^, 72 ± 3 mmolL^–1^, and 94 ± 5 mmolL^–1^; site B, 133 ± 2 mmolL^–1^, 70 ± 4 mmolL^–1^, and 91 ± 6 mmolL^–1^, respectively; *P* > 0.05 in all cases, 6 per site; Figure 1d). The coefficient of variation for each ROI was found to be less than 10% for both sites despite data acquired for different individuals at both sites (medulla, 10% and 5%; cortex, 5% and 4%; and whole kidney, 8% and 5%; for site A and B, respectively).

Because of the high level of consistency within and between subjects, data were pooled and the average sodium concentration values for medulla, cortex, and whole kidney derived for all volunteers (136 ± 7 mmolL^–1^, 72 ± 6 mmolL^–1^, and 93 ± 9 mmolL^–1^, respectively; n = 12). A significant difference in the sodium concentration between all ROIs was found (*P* < 0.05 in all cases). Multilayer segmentation revealed a linear increase in the sodium concentration from cortex to medulla (*R*^2^ = 0.94, *P* < 0.05; Figure 1e).

### Dynamic renal sodium imaging on clinical MRI systems can describe the action of furosemide in the porcine kidney before serum changes

**Figure 2:**
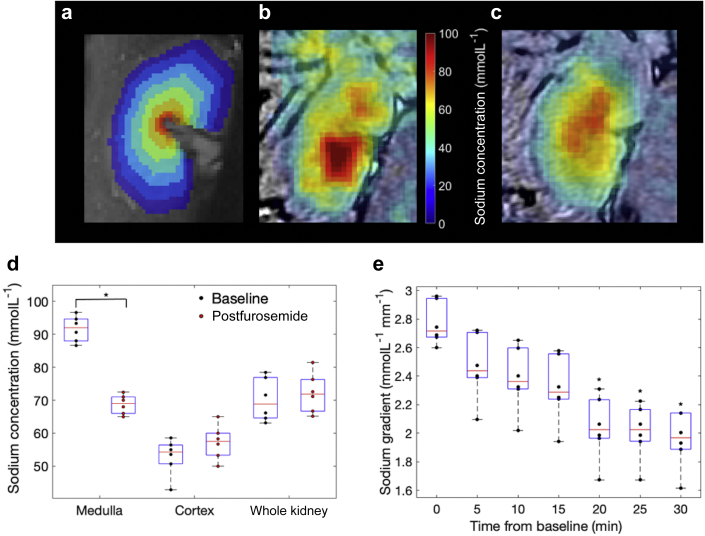
**Porcine sodium imaging.** (**a**) Seven-layer segmentation of the porcine kidney. (**b**) Fused baseline proton-sodium imaging from a different subject demonstrating higher sodium concentration in the medulla compared with the cortex. (**c**) Fused proton-sodium imaging 30 minutes after furosemide from the sample subject as (**b**). (**d**) Sodium concentration derived by manual segmentation results before (black) and 30 minutes after (red) furosemide introduction, revealing a significant decrease in medullary sodium concentration. ∗Significant difference, *P* < 0.05. (**e**) Dynamic changes in the corticomedullary sodium gradient revealing a significant decrease in the corticomedullary sodium gradient 20 minutes after furosemide introduction. ∗Significant difference, *P* < 0.05.

Renal sodium imaging and semiautomated renal segmentation were successfully performed in all porcine exams. Examples of the segmented layers are shown in Figure 2a, and fused ^1^H and ^23^Na concentration images are shown in Figure 2b and c. ROI analysis revealed no significant difference between the sodium results on the 2 different systems (medulla, 90 ± 5 vs. 92 ± 3; cortex, 54 ± 3 vs. 50 ± 3; whole kidney, 70 ± 3 vs. 71 ± 4; *P* > 0.05 in all cases, 3 per group).

Data from both sites were pooled as before, and a significant change in the medulla sodium concentration was observed at 30 minutes after furosemide (Figure 2d; 92 ± 4 vs. 69 ± 3 mmolL^–1^, *P* < 0.05); however, no change occurred in the cortex and whole kidney regions (Figure 2d; 53 ± 5 vs. 57 ± 5 mmolL^–1^ and 70 ± 5 vs. 72 ± 6 mmolL^–1^, respectively; *P* > 0.05). Furthermore, a significant difference in the corticomedullary sodium gradient, in comparison with baseline, was first found at 20 minutes after furosemide introduction (Figure 2e; 2.76 ± 0.15 vs 2.03 ± 0.23 mmolL^–1^mm^–1^, *P* < 0.05).

### Alterations in renal sodium imaging are independent of alterations in blood metabolites

In contrast to the alterations observed in the above experiment, no significant alterations in serum electrolytes were detected at any time point, relative to baseline (baseline vs. 30-minute postfurosemide injection measurements: sodium, 139 ± 2 vs. 139 ± 2 mmolL^–1^; potassium, 103 ± 2 vs. 100 ± 2 mmolL^–1^; chloride, 3.0 ± 0.3 vs. 3.8 ± 0.1 mmolL^–1^; n = 6, *P* > 0.05 in all cases). The maximum change in sodium, potassium, and chloride were 1.4% ± 1.1%, 0.5% ± 1.0%, and –5.0% ± 2.0% mmolL^–1^ at 30 minutes after furosemide. Furthermore, there was no significant correlation between the slope of the corticomedullary gradient and percentage of alterations in serum metabolites (*R*^2^ < 0.1 and *P* > 0.05 in all cases).

### *Ex vivo* renal sodium measurements

**Figure 3:**
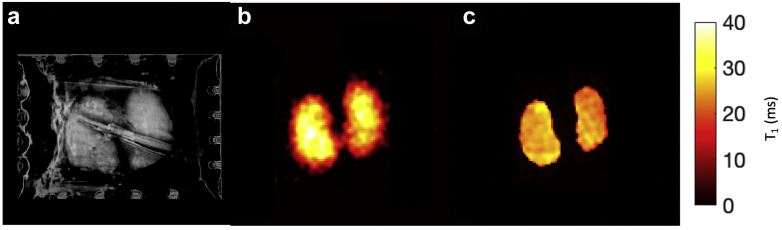
***Ex vivo* porcine kidney measurements.** (**a**) ^1^H imaging of *ex vivo* kidneys (the stripe though both kidneys is an imaging artifact). (**b**) T_1_-weighted image (inversion time = 30 ms) of *ex vivo* kidneys. (**c**) T_1_ map of *ex vivo* kidneys.

*Ex vivo* renal T_1_ mapping was successfully performed on all porcine kidneys (see Figure 3a-c for examples of proton [T_1_ weighted], sodium T_1_-weighted imaging [TI = 30 ms], and sodium T_1_ maps, respectively). The mean T_1_ of the porcine kidney and sodium phantoms was 26 ± 2 ms and 22 ± 2 ms, respectively. Statistical analysis showed no significant difference between these T_1_s, with only a 3% difference in the acquired signal because of T_1_ saturation differences for the TR and flip angle used in this study (*P* > 0.05). A comparison of the *in vivo* and *ex vivo* concentration maps showed very similar sodium distributions, with no significant difference between *in vivo* versus *ex vivo* in cortical (53 ± 5 vs. 54 ± 4 mmolL^–1^, respectively; *P* > 0.05) or medullary (91 ± 5 vs. 84 ± 4 vs mmolL^–1^, respectively; *P* > 0.05) sodium concentration measurements.

Sodium assay results were significantly lower than the *ex vivo* imaging measurements but showed a strong significant linear correlation. ^23^Na-MRI versus assay results in the medulla were 78 ± 9 versus 39 ± 4 mmolL^–1^, respectively, and from the cortex were 45 ± 9 versus 22 ± 4 mmolL^–1^, respectively (*R*^2^ = 0.92, *P* = 0.04; Supplementary Figure S1).

## Discussion

This study demonstrated the potential of renal sodium imaging in both the healthy human and porcine kidney as part of a multisite study. To our knowledge, this is the first time renal ^23^Na-MRI results have been compared between clinical sites. The results show a strong correlation in the quantitative sodium measurements derived from segmented ROIs between individuals and a consistent corticomedullary sodium gradient. Furthermore, this study has shown that dynamic sodium imaging can be performed on a clinical system, revealing alterations in the porcine corticomedullary sodium gradient after the introduction of the diuretic furosemide, whereas blood metabolites remained unchanged. This result demonstrates the power of sodium imaging to understand early physiologic disturbances in renal sodium handling, which may indicate early disease development and therapeutic response, for example in acute kidney injury or chronic kidney disease.^14^

Although ^23^Na-MRI of the brain has been undertaken for a number of years, a number of challenges for body sodium imaging are not present in the brain, for example using gating to compensate for respiratory motion, leading to potential T_1_ saturation differences between organs and concentration phantoms, because of the short TR required for feasible imaging. Furthermore, radiofrequency inhomogeneity across the field of view will induce variations in the sodium signal that are not physiologic in origin. In this study we have demonstrated that the T_1_ of 4% agar phantoms was very similar to the renal system, leading to increased confidence in concentration measures. Our sodium T_1_ results are similar to those published previously, despite differing acquisition sequences.^15^ The difference between porcine and human medullary sodium concentrations may arise from different fluid loading during imaging, because this has been shown to reduce the corticomedullary sodium gradient in previous studies.^9^

This study compared the ^23^Na-MRI with measurements from biopsy samples: Although there was a correlation between *ex vivo* imaging and *ex vivo* chemical analysis, the results from *ex vivo* biochemical analysis of tissue sodium were approximately half those derived from imaging. We hypothesize that this is due to the nature of the tissue preparation where blood was removed from the vascular compartment before sampling, which in turn may have affected the extravascular extracellular compartment. Consequently, the assay was weighted toward the intracellular sodium compartment, whereas imaging combines the signal from both intra- and extracellular compartments. Importantly, the relative ratio of medullary-to-cortical sodium measured using both *ex vivo* sampling and *in vivo* imaging was similar. Future validation of human sodium concentration results presented here with *ex vivo* measurements from human renal tissue samples could be used to reduce any systematic bias in sodium concentration estimates. The quantitative human tissue sodium results presented here are similar to those acquired previously before water loading at a single clinical site (63.5 ± 9 vs. 108 ± 10.9 mmolL^–1^, cortex vs. medulla, respectively).^9^ It is notable that although a transmit radiofrequency (B_1_) correction was performed, a receive B_1_ correction was not. Further correction for receive B_1_ may increase the accuracy of the imaging measurement.

This study suggests the power of using ^23^Na-MRI in further clinical studies, for example chronic kidney disease, acute renal injury, and renal infection, which may result in sodium gradient impairments.^3^^,^^8^^,^^16^^,^^17^ Renal ^23^Na-MRI may provide new information on the pathogenesis of these conditions and a noninvasive method to assess early therapeutic response, which is currently challenging with conventional imaging methods.

## Methods

This report describes a prospective study undertaken between February 2015 and June 2016. Twelve healthy volunteers were recruited, split evenly between sites A (5 men, 1 woman, mean age: 30 ± 2 years) and B (6 men, mean age: 28 ± 5 years) and imaged at 3 T. Written informed consent was obtained from each volunteer, and the study was performed under ethical approval (08/H0311/117). Each volunteer was required to drink 500 mL of water before imaging.

Two groups of 3 Danish domestic pigs were imaged on 2 different MRI scanners (HDx and MR750 at site B; GE Healthcare, Milwaukee, WI) with sodium imaging before and at 5-minute intervals after the introduction of a furosemide bolus (0.5 mg kg^–1^) over 30 minutes. Experiments were carried out in accordance with relevant laws and ethics and under permission from The Animal Experiments Inspectorate in Denmark.

Sodium concentration maps were produced from the imaging data and ROIs drawn to manually segment into the medulla, cortex, and whole kidney.^18^ A semiautomated layer method (see Figures 1c and 2a) was also used to generate 7 and 12 layers within the porcine and human kidneys, because of renal size, respectively.^19^

*Ex vivo* experiments were performed on 4 separate porcine kidneys. The animals were killed by intravenous injection of a pentobarbital overdose. Both kidneys were excised, rinsed in saline (154 mmolL^–1^), and placed in a phantom filled with sodium chloride (154 mmolL^–1^) kept at 37 °C. The phantom with kidneys were placed into the MRI scanner (MR750, site B). Sodium T_1_-weighted imaging was performed and T_1_ maps calculated. Time from when the animals were killed to scan was between 1 and 2 hours. ROIs were drawn in each kidney and the average T_1_ for the renal system calculated. Further ROIs were drawn and used to segment the cortex and medulla of each kidney, and the average renal sodium concentration was calculated.

Twelve biopsy samples were taken: 3 from the medulla and 3 from the cortex from each of 2 of the *ex vivo* porcine kidneys were acquired and the tissue sodium concentration calculated using a sodium assay (Sodium Assay Kit, MAK247; Merck, Kenilworth, NJ) as per the manufacturer’s instructions (full details in Supplementary Methods). The imaging and postprocessing protocol is further detailed in the Supplementary Methods.

### Statistical analysis

Statistical significance was defined at *P* < 0.05 for all analysis, and all statistical calculations were performed in Matlab 2018a (The Mathworks, Natick, MA). Intersite differences between the human ROI segmented sodium concentrations were assessed using Wilcoxon signed-rank tests. Intrasite variations were evaluated with coefficients of variation. After data pooling, Kruskal-Wallis and Wilcoxon signed-rank tests with Bonferroni correction were performed to assess for differences between ROI segmented regions.

The porcine kidney was assessed as above, with a further assessment of difference in the corticomedullary gradient (mM/mm) at each time point by performing Kruskal-Wallis and multiple-comparison corrected Wilcoxon signed-rank tests. Dynamic alterations in serum sodium, potassium, and chloride, relative to baseline, were assessed using a Kruskal-Wallis test and Wilcoxon signed-rank tests and correlated with the renal sodium gradient using a linear fit. Differences in renal and phantom T_1_ were assessed using a Wilcoxon signed-rank test. Differences between the *ex vivo* and *in vivo* renal sodium compartment measurements were compared using a Wilcoxon signed-rank test. The mean sodium concentration in the *ex vivo* biopsy samples was calculated and a linear fit performed to correlate *ex vivo* imaging and *ex vivo* assay results.

## Disclosure

All the authors declared no competing interests.

## References

[1] Jorgensen P.L. (1986). Structure, function and regulation of Na,K-ATPase in the kidney. Kidney Int.

[2] Nielsen P.M., Szocska Hansen E.S., Nørlinger T.S. (2016). Renal ischemia and reperfusion assessment with three-dimensional hyperpolarized 13C,15N2-urea. Magn Reson Med.

[3] Berry M.R., Mathews R.J., Ferdinand J.R. (2017). Renal sodium gradient orchestrates a dynamic antibacterial defense zone. Cell.

[4] Aw M., Armstrong T.M., Nawata C.M. (2018). Body mass-specific Na ^+^ -K ^+^ -ATPase activity in the medullary thick ascending limb: implications for species-dependent urine concentrating mechanisms. Am J Physiol Integr Comp Physiol.

[5] Buerkert J., Martin D., Prasad J. (1981). Role of deep nephrons and the terminal collecting duct in a mannitol-induced diuresis. Am J Physiol Ren Physiol.

[6] Character D., The O.F., Capacity M. (1968). The time course of changes in renal tissue composition during mannitol diuresis in the rat. J Physiol.

[7] Maril N., Margalit R., Mispelter J. (2005). Sodium magnetic resonance imaging of diuresis: spatial and kinetic response. Magn Reson Med.

[8] Maril N., Margalit R., Rosen S. (2006). Detection of evolving acute tubular necrosis with renal 23Na MRI: studies in rats. Kidney Int.

[9] Haneder S., Konstandin S., Morelli J.N. (2011). 23Na MR imaging of the human kidneys at 3 T: before and after a water load. Radiology.

[10] Moon C.H., Furlan A., Kim J.H. (2014). Quantitative sodium MR imaging of native versus transplanted kidneys using a dual-tuned proton/sodium (1H/23Na) coil: initial experience. Eur Radiol.

[11] Zöllner F.G., Konstandin S., Lommen J. (2016). Quantitative sodium MRI of kidney. NMR Biomed.

[12] Maril N., Rosen Y., Reynolds G.H. (2006). Sodium MRI of the human kidney at 3 tesla. Magn Reson Med.

[13] Maril N., Margalit R., Mispelter J. (2004). Functional sodium magnetic resonance imaging of the intact rat kidney. Kidney Int.

[14] Madelin G., Regatte R.R. (2013). Biomedical applications of sodium MRI in vivo. J Magn Reson Imaging.

[15] Gomolka R.S., Ciritsis A., Meier A. (2020). Quantification of sodium T1 in abdominal tissues at 3 T. Magn Reson Mater Phys Biol Med.

[16] Beck F.X., Sone M., Dorge A. (1992). Effect of loop diuretics on organic osmolytes and cell electrolytes in the renal outer medulla. Kidney Int.

[17] David P., Basile M.D., Anderson T.A.S. (2012). Pathophysiology of acute kidney injury. Compr Physiol.

[18] Christensen J.D., Barrère B.J., Boada F.E. (1996). Quantitative tissue sodium concentration mapping of normal rat brain. Magn Reson Med.

[19] Piskunowicz M., Hofmann L., Zuercher E. (2015). A new technique with high reproducibility to estimate renal oxygenation using BOLD-MRI in chronic kidney disease. Magn Reson Imaging.

