# Supplementary material for: Visualization of sodium dynamics in the kidney by magnetic resonance imaging in a multi-site study
Source: Kidney Int. 2020 Nov;98(5):1174–8. doi: 10.1016/j.kint.2020.04.056 (PMC7652549; doi:10.1016/j.kint.2020.04.056)
Supplement: Supplementary File (PDF) [file mmc1.pdf]

Supplementary material

Methods

Imaging protocol

Site A volunteers were imaged on a 3 T GE MR750 system (GE Healthcare, WI). ^{23}Na-MRI was performed at this site with an in-house built flexible surface loop coil. A 3D cones readout was used (3D, hard excitation, pulse width = 0.5 ms, respiratory gated, FOV = 240 mm, TE = 0.5 ms, number of averages (NEX) = 10, number of readouts per volume = 197, number of excitations per gating window= 12, TR = 50 ms, FA = 60 degrees, readout length = 30 ms, acquisition time = approximately 10 min, reconstruction matrix = 96x96x96). A dual flip angle scheme was used to estimate ^{23}Na transmit B_1 maps (acquisition as above, 1 NEX, FA = 30 and 60 degrees)[20]. Proton T_2 weighted imaging was performed using the body coil (fat saturation, respiratory gated, coronal plane, FOV = 380 mm, TR = 6375 ms, TE = 87 ms, MTX = 256x256, slice thickness = 6 mm). Sodium concentration phantoms were placed in the imaging field of view (32 and 80 mmolL^{-1}, 4% agar).

Volunteers at site B were imaged on a 3 T GE HDx system (GE Healthcare, WI). Proton T_2 weighted images of the kidneys were acquired with an 8-channel abdominal array coil (GE Healthcare, Waukesha, WI): fat saturation, breath-hold, coronal plane, FOV = 380 mm, slice thickness = 2 mm, TR = 8652 ms, averages (NEX) = 2, slice spacing = 6 mm, MTX = 256x256, FA = 90 degrees.

Sodium imaging was performed at site B with two Helmholtz loop coils placed anteriorly and posteriorly over the kidneys, protocol as above.

Three pigs were imaged on the above HDx platform and a further three on an MR750 at site B. Pigs were anaesthetised with propofol (0.4 mg/kg/h) and fentanyl (8 µg/kg/h), and ventilated. Arterial blood was sampled from pigs before the baseline scan, and subsequently at each time point.

Pigs were placed supine in the scanner. T_1 weighted ^{1}H imaging was performed using an 8-channel cardiac array (FOV = 380mm, reconstruction matrix = 288x288x192, TR = 43 ms, TE = 1 ms, FA = 12 degrees). Subsequent sodium imaging was performed with a Helmholtz loop coil pair as described above, including respiratory gating, with 5 NEX per volume and imaging acquired in 5 min blocks. Phantoms used were as described above in the site A protocol. IV furosemide (0.5 mgkg^{-1}) was administered after baseline imaging and sodium imaging was performed with 5 min resolution for 30 minutes.

Arterial blood gas was sampled between each dynamic scan, with serum electrolytes (sodium, potassium, and chlorine) quantified.

Ex vivo renal sodium T_1 weighted imaging was performed on four separate porcine kidneys post excision which were placed into a saline (154 $mmolL^{-1}$) filled phantom at 37°C using a Helmholtz loop pair, with the coils placed above and below the phantom in the magnet. The imaging protocol consisted of ^1H scout images (Fast Imaging Employing Steady-state Acquisition, TR = 4 ms, TE = 2 ms, inversion time = 210 ms, flip angle = 65 degrees, slice thickness = 20 mm, slice spacing = 50 mm, averages = 16, FOV = 320mm, matrix = 256x256) and the sodium sequence described above, with the following modifications: TR = 250 ms, inversion preparation (adiabatic hyperbolic secant), inversion times = 20, 22, 26, 30, 34, 42 ms. T_1 maps were calculated fitting a single exponential to data with non-linear least squares fitting in Matlab, fitting for T_1 and the sodium density signal (M_0). In total, four kidneys were imaged. Sodium concentration maps were formed from the M_0 image after T_1 fitting using normal saline (154 $mmolL^{-1}$), and noise (i.e. 0 $mmolL^{-1}$) as reference standards.

Image post-processing and analysis

Regions of interest (ROIs) were drawn on the coronal T_2 images by a researcher, from three central slices in both kidneys, and were used to segment the renal cortex, medulla, and the whole kidney. Image processing and statistical analysis was performed in Matlab (Mathworks, 2017a, Natick, MA). Sodium images underwent B_1 correction before the concentration phantoms were used to create a linear calibration curve, see equation 1, with the sodium zero concentration value taken from the noise present outside of the body in the sodium images[18,20]. ROIs were transferred onto the sodium maps to estimate the average sodium concentration, in the segmented regions, in each subject.

$$\text{Sodium concentration } (mmolL^{-1}) = \frac{Signal^{Voxel} - ConcentrationOffset}{ConcentrationCoefficient} \quad (1)$$

Where the ConcentrationCoefficient and ConcentrationOffset are derived from a linear fit between sodium phantom signal values and a region of noise. $Signal^{Voxel}$is the signal in a given voxel in the imaging slice.

The corticomedullary sodium gradient was defined by segmenting the kidney into concentric layers of equal thickness as previously described[21]. Twelve and seven layers were used for human and porcine segmentation, respectively. Voxels in each layer were averaged across all subjects at each site and combined to form a site-averaged corticomedullary sodium gradient.

After site data pooling the porcine sodium gradient was assessed as above. Dynamic alterations in the gradient were assessed (mM/mm) at each time point.

The % difference between each serum electrolyte at a given time point and baseline was calculated according to equation 3:

$$\%change = 100 \times \left(\frac{baseline_{serumeletrolyte} - timepoint_{serumelectrolyte}}{baseline_{serumeletrolyte}}\right) \quad (3)$$

Where $baseline_{serumeletrolyte}$ is the concentration of a given metabolite (mmolL^{-1}), and $timepoint_{serumelectrolyte}$ is the concentration after administration of furosemide, at a given time point. Data were averaged over the subject group after % change was calculated.

Ex vivo renal analysis was performed by drawing regions of interest around each kidney, and the average T_1 was calculated for all kidneys. Further regions were drawn segmenting the cortex and medulla for each kidney, with the mean sodium concentration in each compartment calculated over all the ROIs.

Further *ex vivo* analysis was performed to validate imaging derived sodium concentration measurements with a sodium assay based on the requirement of sodium ion as a cofactor for the enzymatic activity of β-Galactosidase (Sodium Assay Kit, MAK247, Merck). Twelve biopsy samples were acquired from *ex vivo* kidneys from the medulla (N = 6) and cortex (N = 6) and tissue were processed according to the manufacturer's instructions. Briefly, kidneys were excised and washed in saline (154 mmolL^{-1}) to remove blood from the renal vasculature. Biopsy samples were acquired, and flash frozen in liquid nitrogen, and stored at -80°C for later analysis. After defrosting, samples were homogenised using 10 mmolL^{-1} Dithiothreitol, after which samples were spun for 10 minutes and supernatants taken for spectrophotometric analysis. A ^{23}Na standard curve was set up as per the assay instructions, and sample absorbance at 405 nm recorded to estimate sodium concentration. *Ex vivo* chemical results were correlated with *ex vivo* imaging data using a linear fit in Matlab.

Figures

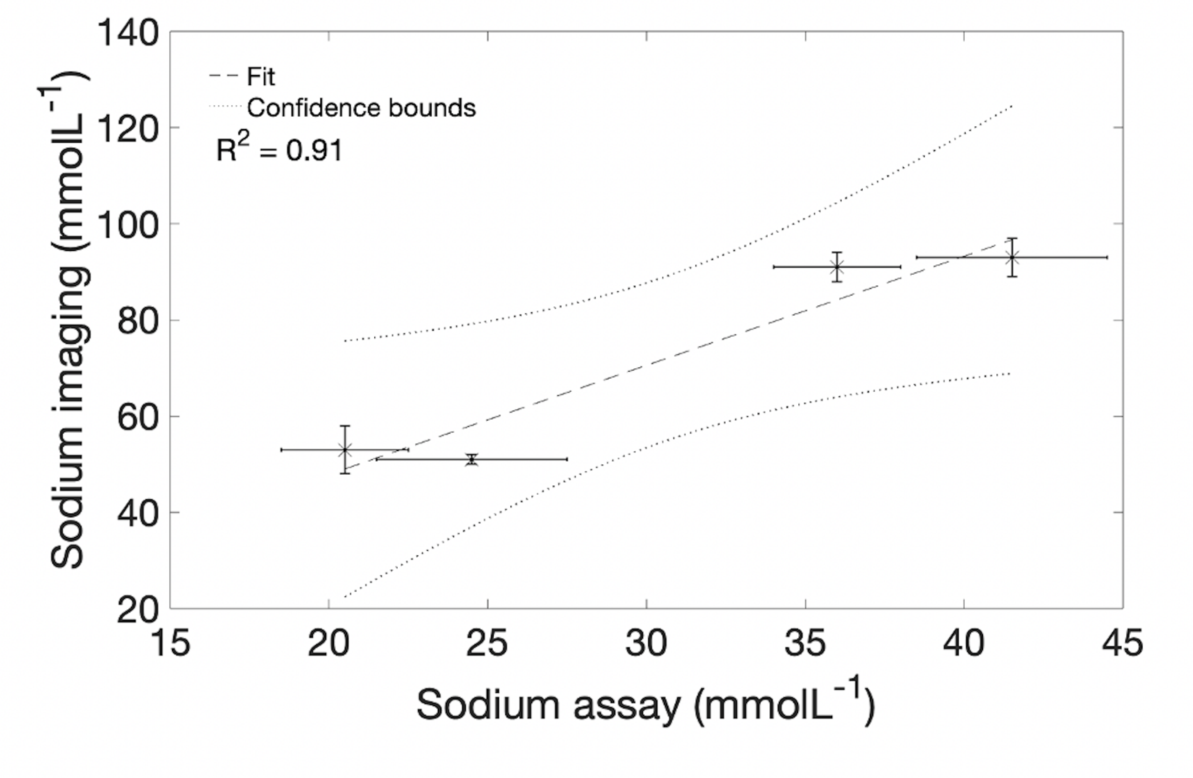

Supplementary figure 1 – *Ex vivo* sodium assay results correlate with *ex vivo* imaging.
